# Reducing the Diabetes Footprint: A Call for Aotearoa New Zealand Diabetic Foot Guidelines

**DOI:** 10.1002/jfa2.70093

**Published:** 2025-11-03

**Authors:** Hannah Jepson, Michele Garrett, Peter A. Lazzarini, Matthew R. Carroll

**Affiliations:** ^1^ Department of Podiatry School of Allied Health Faculty of Health & Environmental Sciences Auckland University of Technology Auckland New Zealand; ^2^ Community and Long Term Conditions Directorate Te Toka Tumai Auckland New Zealand; ^3^ School of Public Health and Social Work Queensland University of Technology Brisbane Queensland Australia; ^4^ Allied Health Research Collaborative Metro North Hospital and Health Service Brisbane Queensland Australia

**Keywords:** amputation, clinical practice guidelines, diabetic foot, health equity, indigenous health

## Abstract

Diabetes‐related foot disease (DFD) affects an estimated 110,000 people in Aotearoa New Zealand (Aotearoa NZ) and is one of the leading causes of the national disease burden. While guideline‐based care has been found to significantly reduce DFD burdens around the world, Aotearoa NZ lacks national DFD guidelines. Instead, Aotearoa NZ clinicians tend to use either international guidelines or fragmented regional pathways of varying quality which result in variability in clinical practice. Given the higher impacts of DFD on Māori and Pacific peoples, and those in socioeconomically deprived or rural areas, national DFD guidelines incorporating Indigenous knowledge are urgently needed in Aotearoa NZ. We call for the urgent development of Aotearoa NZ DFD guidelines and propose methods to co‐develop evidence‐based guidelines integrating clinical expertise with Indigenous perspectives. This approach will enhance consistency, improve health outcomes, and support equitable DFD care in Aotearoa NZ.

## 1 Introduction

Globally, diabetes is estimated to affect 537 million people and be the seventh largest cause of the global disease burden [1]. In Aotearoa New Zealand (Aotearoa NZ), diabetes affects 323,700 people and costs $2.1 billion New Zealand Dollars, with both figures forecast to rise by 50% in 2044 [2, 3]. Diabetes‐related foot disease (DFD) has been reported to be the leading cause of national diabetes disease burdens, yet it typically receives the least diabetes‐related focus [4]. To reduce the rapidly rising national diabetes burden and ensure healthcare sustainability, there is an urgent need for effective, evidence‐based healthcare interventions, particularly for DFD.

## 2 Diabetes‐Related Foot Disease: A Significant Health Challenge For Aotearoa New Zealand

Globally, DFD affects 200 million people and causes over half of the global diabetes disease burden [4]. DFD is defined as disease of the foot in a person with diabetes that includes one or more of the following conditions: peripheral neuropathy, peripheral artery disease, ulcers, infections, and amputations [5]. In Aotearoa NZ 110,000 people are estimated to have some form of DFD, resulting in at least 700 limb amputations each year [6, 7, 8]. Furthermore, the burden of DFD disproportionately affects Māori and Pacific peoples, who experience significantly higher amputation rates [9, 10]. Geographic remoteness and limited access to specialised care further compound these inequities, with amputation rates in the most deprived areas being higher than in the most affluent areas [9, 11].

## 3 Guideline‐Based Care: A Strategy to Reduce Diabetes‐Related Foot Disease Burden

Guideline‐based care for DFD prevention and management has been found to significantly reduces hospitalisation, amputation, and disease burdens around the world [12], whilst also being cost‐saving compared to standard care [13]. Research also shows that combining guideline‐based care models with evidence‐based, trauma‐informed, and culturally safe approaches for Indigenous peoples further improves DFD outcomes [14, 15]. Evidence‐based guidelines do this by supporting healthcare professionals to navigate the increasing volume of evidence, broadening the scope of clinical decision‐making, and emphasising patient involvement [16]. This is crucial given many healthcare professionals lack time and resources to evaluate relevant literature thoroughly, potentially leading to confusion, variation in clinical care and wasted healthcare resources [17].

## 4 The Absence of Diabetes‐Related Foot Disease Burden Guidelines in Aotearoa New Zealand

With the disbanding of the New Zealand Guidelines Group in 2012, clinical management for DFD within Aotearoa NZ is now not supported by locally adapted national guidelines [18]. To try and address this gap, the New Zealand Society for the Study of Diabetes (NZSSD) developed a national foot screening and referral pathway (based on the Scottish Intercollegiate Guidelines), alongside regional community health pathways, which act to support DFD screening, assessment, and management [19]. Although these pathways have been developed to support the integration of guidelines into the NZ health system, for DFD they support only primary care identification and early escalation of care for people with DFD [19]. With DFD being a leading cause of national disease burdens in Aotearoa NZ and worldwide [1], there is an urgent need for guideline‐based care to support the prevention and management of DFD [13].

## 5 The Case for Diabetes‐Related Foot Disease Burden Guidelines in Aotearoa New Zealand

Diabetic foot disease is a complex mix of conditions, typically involving vascular, neurological, musculoskeletal and dermatological pathology, and hence the care of a person with DFD has been reported to potentially involve up to 25 different healthcare professionals [20]. DFD care in practice is also typically provided by a mix of solo clinicians and multidisciplinary healthcare teams. Thus, it is perhaps even more critical for DFD management that guidelines provide consistent processes, language, classification systems, and evidence‐based treatment recommendations for all involved. In Aotearoa NZ it has been identified that international or other national DFD guidelines are often used in clinical practice, such as the International Working Group of the Diabetic Foot (IWGDF) and Diabetes Feet Australia guidelines, with a recent survey indicating these international DFD guidelines were used to inform management by at least half of all podiatrists [21]. Although often robustly developed, international guidelines lack an Aotearoa NZ‐specific context, reducing their ability to guide practitioners in applying recommendations to individual patients in Aotearoa NZ. A recent case investigated by the New Zealand Health and Disability Commissioner highlighted the devastating consequences of fragmented DFD care [22]. In this case, communication failures and inconsistent referral terminology between healthcare professionals resulted in a patient with DFD undergoing multiple amputations before ultimately dying [22]. This case is just one of many that highlights the critical need for standardised clinical protocols and demonstrates the real‐world impact of inadequate or absent guideline implementation [22].

## 6 Developing New Diabetes‐Related Foot Disease Burden Guidelines: A Proposed Plan

Guidelines are more likely to be adopted when there is strong healthcare professional support, a robust evidence base, local context adaption, and implementation tracking systems [23]. However, the cost, time, and expertise required to develop and maintain local guidelines is often substantial.

Australia's experience in developing national DFD guidelines highlight the challenges and potential solutions for developing new national DFD guidelines [24]. Recognising that developing any guidelines from scratch requires at least an estimated $1 million Australian dollars, Diabetes Feet Australia and the Australian Diabetes Society, employed the ADAPTE methodology (systematic adaptation of existing international guidelines), alongside the Grading of Recommendations, Assessment, Development and Evaluation (GRADE) approach to adapt the 2019 IWGDF DFD guidelines to the Australian context [24]. This process took nearly two years, and involved 30 multidisciplinary experts from 7 different healthcare professions, alongside consumer and Aboriginal and Torres Strait Islander expert representatives, to systematically adapt the IWGDF DFD guidelines to the Australian context [25]. These Australian DFD guidelines have been subsequently endorsed by 10 national healthcare professional organisations representing over 10,000 healthcare professional members in Australia [24, 26].

We propose utilising similar processes of adapting contemporary international DFD guidelines to the Aotearoa NZ context. This could involve following a similar method to the Australian experience to develop a specific Aotearoa NZ DFD guideline or alternatively involve forming a trans‐Tasman collaboration with Australian DFD organisations, such as Diabetes Feet Australia, and partnering with Māori, Aboriginal, and Torres Strait Islander Peoples to develop a joint Aotearoa NZ/Australian DFD guideline addressing the contexts of both countries. Regardless, any approach should establish DFD expert panels comprising multi‐disciplinary experts, Indigenous peoples, researchers, and consumer representatives and utilise evidence‐based guideline development approaches, such as ADAPTE and GRADE. Partnering with Māori and Aboriginal and Torres Strait Islander Peoples in particular means that Indigenous perspectives are integral throughout the development process of any future guideline and help address the disproportional burden of DFD in Indigenous Peoples.

With the next IWGDF DFD guidelines due in 2027, this presents an optimal window for collaborative preparation, potentially allowing Australia to refresh their 2021 guidelines whilst Aotearoa NZ develops their inaugural national guidelines. Such a collaborative methodology could systematically assess each IWGDF recommendation through shared bicultural expertise, with parallel development processes producing either a joint or distinct guideline for each country. Any Aotearoa NZ guideline would incorporate Māori health frameworks, unique healthcare delivery models, and population‐specific needs, whilst Australian guidelines would reflect their established contextual factors and Aboriginal and Torres Strait Islander Peoples perspectives. This shared approach could help share the estimated $250,000 development costs whilst ensuring guidelines reflect the distinct needs of both nations and Indigenous populations [26].

## 7 Conclusion: Shaping the Future of Diabetes‐Related Foot Disease Care in Aotearoa New Zealand

Aotearoa NZ faces a very large and rapidly increasing DFD burden. National DFD guidelines are powerful tools for reducing national DFD burdens, addressing local healthcare system challenges, preventing and managing local DFD patients, supporting advocacy and enabling cost‐effective, accessible, person‐centred care. Currently, Aotearoa NZ healthcare professionals often use international guidelines to guide DFD care which lack contextualisation and co‐design with Māori knowledge perspectives. Considering the development of country‐specific guidelines are costly, we suggest a trans‐Tasman partnership approach with Australia in the development of future DFD guidelines. Such a trans‐Tasman collaboration could share clinical expertise, Indigenous Peoples knowledges, and costs to produce evidence‐based guidelines representing the unique needs of the Aotearoa NZ health system and people.

## Author Contributions

**Hannah Jepson:** conceptualisation (lead), writing – original draft, writing – review and editing. **Michele Garrett:** conceptualisation, writing – review and editing. **Peter A. Lazzarini:** conceptualisation, writing – review and editing. **Matthew R. Carroll:** conceptualisation, writing – review and editing. All authors approved the final manuscript.

## Funding

The authors have nothing to report.

## Conflicts of Interest

The authors declare no conflicts of interest.

## Data Availability

Data sharing is not applicable to this article as no new data were created or analysed in this study.
